# Phosphorus in Plants

**Published:** 1860-10

**Authors:** 


*Phosphorus in Plants.*—M. B. Corenwinder read lately before the French Academy of Sciences a *resume* of his studies on this subject. Young plants give ashes rich in phosphoric acid; but after maturity the grain, or fruit, stalks, or leaves contain but a small proportion. Phosphoric acid in plants is found in close cimbination with nitrogenous matters. The organs of the destitute of nitrogen and not required for its alimentation are also destitute of phosphates; but the pollen of flowers and the spores of cryptogamia contain a considerable portion of phosphoric acid. Marine plants growing on rocks also contain much phosphate.—*Lancet.*

